# Online multifactorial prevention programme has no effect on the number of running-related injuries: a randomised controlled trial

**DOI:** 10.1136/bjsports-2018-099744

**Published:** 2019-04-06

**Authors:** Tryntsje Fokkema, Robert-Jan de Vos, John M van Ochten, Jan A N Verhaar, Irene S Davis, Patrick J E Bindels, Sita M A Bierma-Zeinstra, Marienke van Middelkoop

**Affiliations:** 1 Department of General Practice, Erasmus MC, University Medical Center, Rotterdam, The Netherlands; 2 Department of Orthopaedics and Sports Medicine, Erasmus MC, University Medical Center, Rotterdam, The Netherlands; 3 Spaulding National Running Center, Department of Physical Medicine and Rehabilitation, Harvard Medical School, Cambridge, Massachusetts, USA

**Keywords:** Running, injury prevention, prevention

## Abstract

**Objective:**

To examine the effect of a multifactorial, online injury prevention programme on the number of running-related injuries (RRIs) in recreational runners.

**Methods:**

Adult recreational runners who registered for a running event (distances 5 km up to 42.195 km) were randomised into the intervention group or control group. Participants in the intervention group were given access to the online injury prevention programme, which consisted of information on evidence-based risk factors and advices to reduce the injury risk. Participants in the control group followed their regular preparation for the running event. The primary outcome measure was the number of self-reported RRIs in the time frame between registration for a running event and 1 month after the running event.

**Results:**

This trial included 2378 recreational runners (1252 men; mean [SD] age 41.2 [11.9] years), of which 1196 were allocated to the intervention group and 1182 to the control group. Of the participants in the intervention group 37.5% (95% CI 34.8 to 40.4) sustained a new RRI during follow-up, compared with 36.7% (95% CI 34.0 to 39.6) in the control group. Univariate logistic regression analysis showed no significant difference between the intervention and control group (OR 1.08; 95% CI 0.90 to 1.30). Furthermore, the prevention programme seemed to have a negative impact on the occurrence of new RRIs in the subgroup of runners with no injuries in the 12 months preceding the trial (OR 1.30; 95% CI 0.99 to 1.70).

**Conclusion:**

A multifactorial, online injury prevention programme did not decrease the total number of RRIs in recreational runners.

**Trial registration number:**

NTR5998.

## Introduction

Running is a sport that is frequently practised and is growing in popularity.^1^ In the Netherlands, about 2 million people performed running regularly in 2014, which is about 12.5% of the Dutch population.^2^ Regular running has many positive effects on both physical and mental health and is an efficient way to improve physical fitness.^3^ A main drawback, however, is the high number of musculoskeletal injuries among runners.

The injury proportions in runners vary between 3.2% and 84.9%, with novice runners having the highest injury proportion and cross-country runners having the lowest proportion.^4^ Survey data suggest that the incidence of running-related injuries (RRIs) has increased over the last years from 4.8 RRIs per 1000 running hours in 2011 up to 6.1 RRIs per 1000 running hours in 2014 in the Netherlands.^2^ In order to prevent future injuries, several studies have aimed to identify risk factors for RRIs. These studies have identified a variety of risk factors, including overweight, a high weekly running distance, a low running cadence and running on outworn shoes.^5–8^ However, the risk factors for RRIs are not uniform between studies.^9–11^ A systematic review showed, for example, that a higher age was identified as a risk factor for RRIs in four studies, while it was a protective factor for RRIs in two other studies.^9^ Only a previous injury is a consistent and frequently identified risk factor for RRIs,^9–11^ which emphasises the need for primary injury prevention measures in runners.

So far, only a few randomised controlled trials (RCTs) have investigated the effects of injury prevention measures in runners.^12–17^ Most of these RCTs targeted one specific risk factor for RRIs. For example, Bredeweg *et al* performed an RCT aiming to modify the risk factor ‘absence of experience with sporting activities with axial loading’.^13^ They offered novice runners a preconditioning training programme with walking and hopping exercises, but this training programme had no effect on the number of RRIs. Also in other RCTs on RRI prevention, no effect on the number of RRIs was found.^12 16 18^ This may be related to the fact that these RCTs targeted only one risk factor for RRIs, while the cause of RRIs seems to be multifactorial.^10 12^ Therefore, the aim of this study was to examine the effect of a multifactorial, online injury prevention programme on the number of RRIs in recreational runners.

## Methods

### Trial design

The INSPIRE trial (INtervention Study on Prevention of Injuries in Runners at Erasmus MC) is a randomised controlled trial with a minimum follow-up of 3 months. A detailed study protocol has been published elsewhere (https://bmjopensem.bmj.com/content/3/1/e000265).^18^ The INSPIRE trial was funded by the Netherlands Organization for Health Research and Development (ZonMW, 536001001) and was performed in collaboration with Golazo Sports, an organisation of large running events in the Netherlands.

### Participants

Potential participants of the INSPIRE trial were runners who registered for one of three large running events in the Netherlands in 2017. These running events included the NN City Pier City The Hague (5, 10 and 21.1 km), NN Marathon Rotterdam (10.55 and 42.195 km) and the LadiesRun Rotterdam (5, 7.5 and 10 km). During the online registration for the running events, the runners were asked if they were willing to participate in the INSPIRE trial. Contact information of the interested runners was sent to the researchers. Runners who met the inclusion criteria (18 years or older, registration at least 2 months before the running event, knowledge of the Dutch language, and access to the internet and email) received more information about the INSPIRE trial through email. If they were still interested in participation, they could immediately provide digital informed consent and complete the baseline questionnaire.

### Randomisation and follow-up

After completing the baseline questionnaire, participants were randomised into either the intervention or control group, using a computer-generated randomisation list with blocks of 10. The randomisation list was developed by an individual who is not part of the research team. The participants were enrolled and assigned to the interventions by a member of the research group.

Participants allocated to the intervention group were given access to an online injury prevention programme. Participants in the control group were informed about their allocation into the control group and consequently followed their regular preparation for the running event. All participants received three follow-up questionnaires during the study period; 2 weeks before the running event they registered for, 1 day after the running event and 1 month after the running event. The participants received additional monthly reminders about the study per email. For the participants in the intervention group, these reminders included a repetition of one of the topics in the injury prevention programme. To improve adherence to the intervention, these reminders also included a link to the intervention website. For the control group, the reminders contained an update of the progress of the INSPIRE trial (eg, information on the number of participants that had been included) or general information on epidemiology of RRIs. Depending on the moment of registration, the participants received maximal five reminders.

### Interventions

The injury prevention programme was developed by means of an extensive literature search and aimed to modify evidence-based risk factors for RRIs. The prevention programme was presented on a website that could only be accessed with a username and password, which were provided to the participants in the intervention group through email. We instructed the participants to keep these data strictly personal. The website contained information on four main topics: personal factors (age, weight, previous injuries and running experience), training (running distance, frequency, surface, overtraining and stretching), biomechanics (cadence and foot landing) and equipment (footwear, orthotics and the use of running shoes). Different versions of the prevention programme for novice and experienced runners were available. Details of the injury prevention programme can be found elsewhere.^18^ Participants in the intervention group had unlimited access to the website. The runners were expected to work autonomously with the website. They were encouraged to read the information they thought was relevant to them and apply this in their training. It was not logged how many times individual runners accessed the site.

### Measurements

The baseline questionnaire consisted of five sections (demographics, training, running events, lifestyle and previous RRIs). The items of these sections are shown in table 1. The follow-up questionnaires informed on RRIs during follow-up and the use of the prevention programme. The items of the follow-up questionnaires are shown in table 1.

**Table 1:** Items of the questionnaires of the INSPIRE trial

Questionnaire	Section	Items
Baseline questionnaire	Demographics	Sex
		Date of birth
		Height (cm)
		Weight (kg)
	Training	Running experience (years)
		Average running frequency over last month (times per week)
		Average running time over last month (min/week)
		Average running distance over last month (km/week) Average training speed over last month (min/km)
		Types of training
		Endurance training (%)
		Interval training (%)
		Exercises (%)
		Membership of athletic association (yes/no)
	Running events	Previous participation in running events (yes/no)
		Average participations in running events per year
	Lifestyle	Current smoking (yes/no)
		Average alcohol consumption (glasses per week)
	Previous running-related injuries*	Running-related injury in previous 12 months (yes/no)
		Location of running injury (lower back/buttock/hip/groin/ventral thigh/dorsal thigh/knee/shin/calf/Achilles tendon/ankle/foot/toe)
		Still suffering running injury (yes/no)
Follow-up questionnaires	Existing running-related injuries*	Still suffering running-related injury that was already indicated in previous questionnaire (yes/no)
		Location of existing running injury (lower back/buttock/hip/groin/ventral thigh/dorsal thigh/knee/shin/calf/Achilles tendon/ankle/foot/toe)
	New running-related injuries*	New running-related injury since filling in previous questionnaire (yes/no)
		Location of new running injury (lower back/buttock/hip/groin/ventral thigh/dorsal thigh/knee/shin/calf/Achilles tendon/ankle/foot/toe)
	Injury prevention programme†	Read injury prevention programme (yes/no)
		If yes, which topic(s) (personal factors/training/biomechanics/equipment)
		Used injury prevention programme (yes/no)
		If yes, which topic(s) (personal factors/training/biomechanics/equipment)

*Participants could list multiple injuries.

†This section was only in the follow-up questionnaires for the intervention group.

### Outcomes

The primary outcome measure of this study was a self-reported RRI between the moment of registration and 1 month after the running event. To avoid confusion, a definition of an RRI was provided to the participants. An RRI was defined as an injury of the muscles, joints, tendons and/or bones in the lower back or lower extremities (hip, groin, thigh, knee, leg, ankle, foot and toes) that was caused by running. Furthermore, one of the following criteria had to be met:

1. The injury was severe enough to cause a reduction in running distance, speed, duration or frequency for at least 1 week.
2. The injury led to a visit of a doctor and/or physiotherapist.
3. Medication was necessary to reduce symptoms as a result of the injury.

The location of the injury was a secondary outcome measure.

### Sample size

Based on a recent systematic review among a mixed population of long-distance runners, an injury incidence of 16% was expected in the control group.^4^ A 10.9% injury incidence has been reported in a study on novice runners with a comparable follow-up time.^19^ Based on these studies, we estimated that 14% of the participants would sustain an injury during follow-up. With a risk difference of 5% (this means a reduction of 90 000 injuries in the Netherlands), 0.05 significance level (one-sided testing) and a power of 80%, a total of 1006 runners had to be included in the analyses to detect a relevant difference in RRIs. Taking a loss to follow-up of 10% into account, at least 1106 participants had to be included in this trial.

### Statistical analyses

Descriptive statistics were calculated for all variables. Consistent with the CONSORT statement, an intention-to-treat analysis was performed. Injury proportions with corresponding 95% CIs were calculated for the whole group and for the intervention and control group separately. We determined the injury proportions by calculating the percentages of participants who indicated a new RRI in one or more of the follow-up questionnaires. To correct for errors, we checked whether participants who indicated they still suffered an existing RRI indeed filled in an RRI on the same location in the previous questionnaire. If not, the RRI was interpreted as a new RRI. Also for RRIs of which participants indicated to be new, we checked whether the participants did not fill in this RRI in the previous questionnaire. If they did, this RRI was not regarded as a new RRI. The injury proportions of the intervention and control group were compared by calculating the difference in percentages with 95% CI between the injury proportions. Additionally, ORs with 95% CI were calculated using univariate logistic regression analysis. Also, the risk ratios with 95% CI were calculated. Finally, adjusted analysis including potential confounders (age, body mass index [BMI] and earlier injury) was performed with multivariate logistic regression analysis.

The number of injured runners per location and the percentages of the total number of participants were determined for the intervention and control group separately. For further analyses, the injury locations were divided into five groups: lower back, buttock/hip/groin, upper leg/knee, lower leg (shin/calf/Achilles tendon/ankle) and foot/toe. Predefined subgroup analyses were performed for sex, running experience (≤1 year/>1 year running experience), distance of running event, earlier RRI in previous 12 months and for the five groups of injury locations separately.^18^ Analyses were performed in SPSS Statistics V.21 and p values ≤0.05 were regarded as statistically significant.

## Results

### Participants

**Figure 1:**
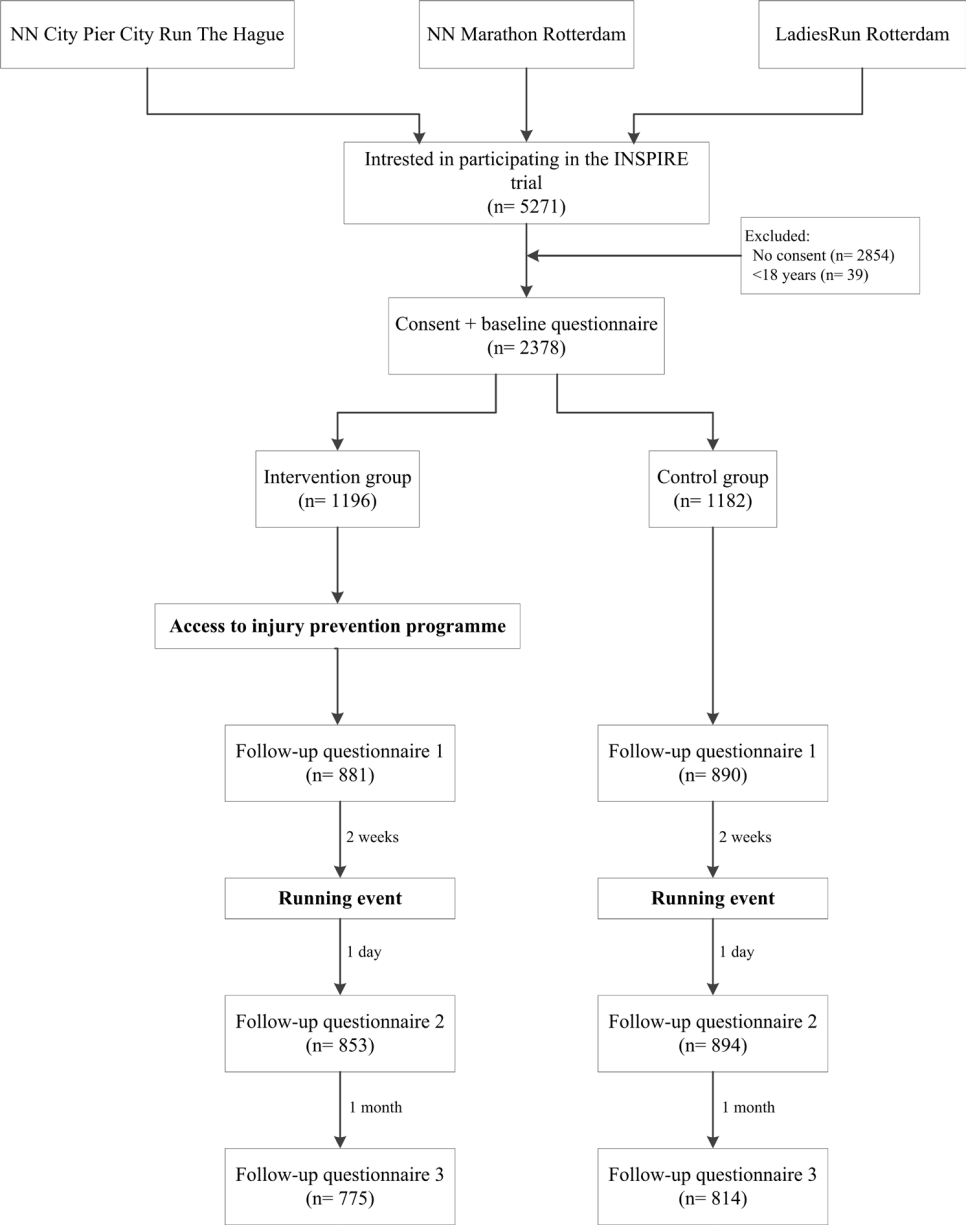
Flowchart of the INSPIRE trial.

Data collection for the INSPIRE trial started in October 2016 and was finalised in August 2017. In total, 5271 runners indicated that they were interested in participation in the INSPIRE trial when they registered for one of the running events, of which 2378 runners were included in the trial (figure 1). After randomisation, 1196 participants were allocated to the intervention group and 1182 participants to the control group. At baseline, the participants were on average 41.2 (SD 11.9) years old and the majority (52.6%) was male (table 2). A total of 52.1% of the participants reported an RRI in the 12 months before inclusion and 22.7% of the participants still suffered an RRI at baseline. There were no significant differences in baseline characteristics between the intervention and control group.

**Table 2:** Baseline characteristics

	All participants		Intervention group		Control group	
	N	%/Mean (SD)	N	%/Mean (SD)	N	%/Mean (SD)
N	2378		1196	50.3%	1182	49.7%
Demographics						
Gender (male)	1252	52.6%	623	52.1%	629	53.2%
Age (years)		41.2 (11.9)		41.0 (11.7)		41.4 (12.0)
BMI (kg/m^2^)		23.7 (2.9)		23.6 (2.9)		23.7 (2.8)
Training						
Running experience (years)		6.5 (7.8)		6.6 (7.9)		6.4 (7.8)
Running frequency per week		2.5 (1.5)		2.5 (1.5)		2.5 (1.4)
Running time per week (hours)		3.1 (3.3)		3.1 (3.3)		3.1 (3.3)
Running distance per week (km)		22.2 (19.0)		22.3 (19.0)		22.1 (19.0)
Training speed (min/km)		6:04 (1:07)		6:03 (1:07)		6:05 (1:07)
Type of training (%)						
Endurance training		68.6 (24.1)		68.5 (24.2)		68.8 (23.9)
Interval training		23.9 (20.9)		24.2 (21.2)		23.6 (20.6)
Exercises		7.4 (10.4)		7.3 (9.7)		7.8 (11.0)
Member of athletic association (yes)	715	30.1%	352	29.4%	363	30.7%
Running event						
Distance registered for:*						
5/7.5 km	139	5.8%	75	6.3%	64	5.4%
10/10.55 km	905	38.1%	440	36.8%	465	39.3%
Half-marathon	711	29.9%	367	30.7%	344	29.1%
Marathon	625	26.3%	317	26.5%	308	26.1%
Participated in a running event before (yes)	2168	91.2%	1092	91.3%	1076	91.0%
Average participations per year		4.2 (5.1)		4.2 (4.7)		4.2 (4.9)
Lifestyle						
Smoking (yes)	107	4.5%	57	4.8%	50	4.2%
Alcohol use (glasses per week)		4.2 (4.8)		4.1 (4.7)		4.3 (4.9)
Previous RRIs						
Previous RRI in previous 12 months (yes)	1238	52.1%	611	51.1%	627	53.0%
Reported RRI at baseline (yes)	540	22.7%	281	23.5%	259	21.9%

*Running distance was missing for two participants, while two participants registered for two and one participant for three running distances of one running event.

BMI, body mass index; RRI, running-related injury.

### Injuries during follow-up

Mean (SD) follow-up duration was 4.5 (1.6) months and 81.1% of the participants completed at least one of the follow-up questionnaires, while 60.0% completed all follow-up questionnaires (figure 1). In total, 28.4% of all follow-up questionnaires were not completed. The majority of the participants in the intervention group (62.7%) indicated that they read at least one topic of the injury prevention programme, of whom 8.2% read one topic, 11.0% read two topics, 4.7% read three topics and 38.8% read all four topics. Also, 44.1% of the participants indicated they applied the information of at least one topic into their training. During follow-up, 883 participants (37.1%, 95% CI 35.2 to 39.1) sustained 1483 new injuries (table 3). The injury proportion for the intervention group was 37.5% (95% CI 34.8 to 40.4) and 36.7% (95% CI 34.0 to 39.6) for the control group, with no significant difference between groups (OR 1.08; 95% CI 0.90 to 1.29) (table 2). In both the intervention and control groups, most injuries were in the knee (10.8% and 12.5%, respectively), calf (6.9% and 6.3%, respectively) and foot (5.9% and 5.8%, respectively) (online appendix 1). Analyses of the clustered injury locations showed no significant differences between the intervention group and control group (table 3). The multivariate logistic regression analysis adjusting for main potential confounders (age, BMI and earlier RRI) showed no difference between study groups (OR 1.08; 95% CI 0.90 to 1.30). Subgroup analyses showed no significant differences in the injury proportions between the intervention and control group when divided by the distance of the running event, sex, running experience or an RRI in the 12 months before the trial (table 4).

10.1136/bjsports-2018-099744.supp1 Supplementary data

**Table 3:** Total number of injuries and number of injured runners per clustered injury locations and the differences between the intervention group (n=1196) and control group (n=1182) and results of univariate logistic regression analysis for the effect of study group on the injury risk and the risk ratio of the intervention group

	Intervention group		Control group		Difference	OR (95% CI)*	Risk ratio (95% CI)
	N	%	N	%	% (95% CI)		
Participants reporting new injuries during follow-up (yes)	449	37.5	434	36.7	0.8 (−3.1 to 4.8)	1.08 (0.90 to 1.29)	1.02 (0.92 to 1.14)
No of new injuries	736		747				
Clustered injury locations							
Lower back	49	4.1	48	4.1	0.0 (−1.6 to 1.7)	1.03 (0.68 to 1.55)	1.01 (0.68 to 1.49)
Buttock/hip/groin	110	9.2	104	8.8	0.4 (−2.0 to 2.8)	1.07 (0.81 to 1.43)	1.05 (0.81 to 1.35)
Upper leg/knee	171	14.3	200	16.9	−2.6 (−5.6 to 0.36)	0.83 (0.66 to 1.04)	0.85 (0.70 to 1.02)
Lower leg	191	16.0	178	15.1	0.9 (−2.1 to 3.9)	1.10 (0.88 to 1.38)	1.06 (0.88 to 1.28)
Foot/toe	78	6.5	75	6.3	0.2 (−1.9 to 2.2)	1.05 (0.76 to 1.46)	1.03 (0.76 to 1.40)

*Control group is reference.

**Table 4:** Results of the subgroup analyses (injury proportions for the intervention and control group and results of univariate logistic regression analysis for the effect of study group on the injury risk and the risk ratio of the intervention group)

	N		Injury proportion (%)		Difference (%) (95% CI)	OR (95% CI)*	Risk ratio (95% CI)
	Intervention group	Control group	Intervention group	Control group			
Distance running event†‡							
Marathon	317	308	41.3	41.2	0.1 (−7.8 to 8.0)	0.97 (0.67 to 1.39)	1.00 (0.83 to 1.20)
Half-marathon	367	344	42.2	38.1	4.2 (−3.3 to 11.5)	1.21 (0.88 to 1.66)	1.11 (0.93 to 1.32)
10/10.55 km	439	464	32.3	33.8	−1.5 (−7.8 to 4.8)	1.02 (0.76 to 1.37)	0.96 (0.79 to 1.15)
5/7.5 km	73	64	28.8	28.1	0.7 (−15.4 to 16.4)	1.13 (0.52 to 2.48)	1.02 (0.60 to 1.74)
Sex							
Male	623	629	39.2	38.3	0.9 (−4.7 to 6.3)	1.10 (0.86 to 1.41)	1.02 (0.89 to 1.18)
Female	573	553	35.8	34.9	0.9 (−4.8 to 6.6)	1.06 (0.81 to 1.38)	1.03 (0.88 to 1.20)
Running experience§							
≤1 year	224	211	36.6	39.3	−2.7 (−12.1 to 6.6)	0.90 (0.58 to 1.38)	0.93 (0.73 to 1.18)
>1 year	969	964	37.8	36.3	1.5 (−2.9 to 5.8)	1.12 (0.92 to 1.36)	1.04 (0.93 to 1.17)
Earlier injury¶							
Yes	611	627	43.9	44.7	−0.8 (−6.4 to 4.8)	0.92 (0.72 to 1.19)	0.98 (0.87 to 1.11)
No	585	555	30.9	27.7	3.2 (−2.2 to 8.6)	1.30 (0.99 to 1.70)	1.12 (0.93 to 1.34)

*Control group is reference.

†Running event is missing for 2 participants.

‡The four participants who registered for multiple distances of one running event were assigned to the longest distance they registered for.

§Running experience is missing for 10 participants.

¶Running injury in year before INSPIRE trial.

## Discussion

This study aimed to reduce running injuries in recreational runners by providing online advice on modifying known risk factors. This multifactorial, easy accessible prevention programme did not decrease the overall number of RRIs in recreational runners. Neither were any differences found in any of the predefined subgroups of runners.

In contrast to previous trials, targeting one single risk factor only, this study investigated the effect of a multifactorial injury prevention programme in runners.^12 13 18^ However, this multifactorial programme did not reduce the overall number of RRIs. This result seems opposite to the effects of multicomponent prevention programmes in team sports (eg, floorball and soccer) that have shown to be effective.^20–22^ One large difference with these types of sports is that runners tend to train individually and often without a trainer or coach. Therefore, the runners were offered an online programme from which they could extract the information of their interest. Almost two-thirds (62.7%) of the participants in the intervention group indicated that they read at least one topic of the prevention programme and 44.1% indicated that they also applied the information into their training. This relatively low engagement rate may have influenced the results. The injury prevention programme was designed to be implementable in large populations of runners. However, the fact that about one-third of the participants did not read any topics of the prevention programme reflects the feasibility of the prevention programme. It may indicate that the participants had problems to extract the relevant information and to apply this into their usual training sessions or may be associated with the attractiveness of the programme. Perhaps runners need more personalised information or more directed practical information (eg, detailed day-to-day training schedules) on injury prevention. Furthermore, stationary websites may no longer be engaged well with and mobile applications might be more successful.^23^ Future analyses and research should therefore focus on the effects of compliance and the feasibility and effectiveness of these types of interventions offered to runners.

With the participants in the intervention group, there was a trend towards less injuries (2.6%) in the upper leg/knee than participants in the control group. In contrast, runners in the intervention group showed a trend to report more injuries in the calf, Achilles tendon, ankle and foot. It is possible that this may be related to the information presented on biomechanics in the injury prevention programme. This section included information regarding forefoot striking resulting in reduced impact forces on the knee and thereby potentially reducing the chance on a knee injury.^24–26^ However, a transition to a forefoot strike increases the loading on the lower leg and foot and may increase the injury risk in these areas.^27 28^ To prevent this, a training programme aimed at strengthening the foot and calf for the transition to a forefoot strike and minimalistic shoes was included in the injury prevention programme.^27^ This training programme also included a gradual progression in the use of a forefoot strike and minimalistic shoes. It is therefore interesting to observe that participants in the intervention group who indicated that they used the biomechanics section reported significantly more lower leg injuries during follow-up than participants in the control group (OR 1.74; 95% CI 1.28 to 2.37) (online appendix 2). It can be hypothesised that these runners used the information from the prevention programme, consequently changed their stride pattern and got injured. This may suggest that changing to a forefoot strike may not be an effective way to prevent RRIs or that the way the training programme and information on stride pattern was offered is not optimal in order to prevent the injuries, also in the lower leg, and might even be harmful to the runners when applied with these methods. Therefore, we suggest not to provide advices on biomechanics if no personal guidance (eg, from a physiotherapist) is available.

10.1136/bjsports-2018-099744.supp2 Supplementary data

The adjusted logistic regression analysis showed that adjustment for main risk factors (age, BMI and previous RRIs) had no influence on the overall effect of the prevention programme (OR 1.08, 95% CI 0.90 to 1.30). This analysis also showed that an RRI in the 12 months before the study was the only factor with a significant effect on the occurrence of new RRIs (OR 2.21; 95% CI 1.84 to 2.65). The majority of the new RRIs (76.6%) occurred at a different location than the previous RRI. This showed again that runners with an RRI in the past have a higher chance of sustaining a new RRI, regardless of the location of the RRI.^9–11^ The subgroup analyses also showed a trend towards more RRIs in the intervention group in runners who did not have an RRI in the 12 months preceding the trial (OR 1.30; 95% CI 0.99 to 1.70). This may suggest that offering injury prevention measures to runners not prone to injuries may result in more new-onset injuries. Possibly these runners already ran and trained in the right way and therefore changing something resulted in injuries. Furthermore, runners who suffered an RRI in the previous 12 months appeared to be more interested in injury prevention. Additional analyses showed that significantly more participants who suffered an RRI in the 12 months before the study indicated that they read at least one of the topics of the intervention programme compared with those without an RRI in the past 12 months (65.6% vs 59.7%, p=0.033). Based on the aforementioned information, injury prevention advices should possibly be geared towards the runner’s RRI history. For example, runners with a history of Achilles tendinopathy may benefit from limiting exposure to running on soft surfaces.^29^ However, more research on tailored programmes is necessary. Furthermore, we suggest that future prevention studies on RRIs should specifically aim at runners with an RRI in the past.

### Strengths and limitations

A strength of the current study is the large sample size. With 2378 participants, it is the largest RCT on RRI prevention so far. Also, the loss to follow-up was relatively low; more than 80% of the participants filled in at least one of the follow-up questionnaires. A limitation of this study is that we had only little insight in the use of the online injury prevention programme. Self-registered information on the use of the prevention programme was collected. It would have been more accurate if the exact use per participant could have been retrieved from the personal visitors statistics of the website. Another limitation is that the RRIs were self-diagnosed, which may have influenced the number of RRIs and the accuracy of the RRIs reported. Also, we had no insight in the severity and impact of the reported RRIs. Furthermore, the definition of an RRI was slightly different from the consensus definition proposed by Yamato *et al*.^30^ We did not use this definition, as it was not available at the time we designed this study in 2015. We based our definition on methods used in previous prospective trials^19 31^ and due to our randomised study design, this chosen definition will not have affected our primary outcome. Finally, in our protocol, we intended to perform multiple imputation when more than 5% of the data were missing.^18^ Main outcome data during follow-up were missing in 28.4% of the cases. The imputation of an RRI during follow-up had no effect on the main outcome (OR 1.16; 95% CI 0.93 to 1.44). We therefore decided to report the outcomes without the imputation.

## Conclusion

A multifactorial, online injury prevention programme offered to recreational runners who registered for a running event was not effective in the prevention of RRIs. We hypothesise that this may be related to the way the information on injury prevention was presented to the runners. Perhaps runners need more personalised information or more directed practical information on injury prevention. Furthermore, it may be related to the heterogeneity in the study population, especially in previous injuries. It is again shown that runners who had an RRI before had a higher chance to sustain a new RRI. Furthermore, the prevention programme seemed to have a negative impact on the occurrence of new RRIs in the subgroup of runners with no injuries in the 12 months preceding the trial. Therefore, future studies on running injury prevention measures may specifically aim at this high-risk group of runners who had an RRI before.

**What are the findings?**

- A multifactorial, online injury prevention programme was not effective in the prevention of running-related injuries (RRIs) in recreational runners.
- The prevention programme seemed to have a negative impact on the occurrence of new RRIs in the subgroup of runners with no injuries in the 12 months preceding the trial.
- The information on biomechanics seemed to have a negative effect on the occurrence of new RRIs.

**How might it impact on clinical practice in the future?**

- A multifactorial, online injury prevention programme is not effective in recreational runners.
- Injury prevention measures should possibly be aimed at runners with a history of RRIs.
- Advices on biomechanics should possibly not be given through a website.
