# Supplementary material for: Online multifactorial prevention programme has no effect on the number of running-related injuries: a randomised controlled trial
Source: Br J Sports Med. 2019 Apr 6;53(23):1479–85. doi: 10.1136/bjsports-2018-099744 (PMC6900232; doi:10.1136/bjsports-2018-099744)
Supplement: Supplementary data [file bjsports-2018-099744supp001.pdf]

Number of injured runners per anatomical side and the differences between the intervention group (n=1196) and control group (n=1182)

Injury location	All participants		Intervention group		Control group		Difference	OR (95% CI) [a]
	N	%	N	%	N	%	% (95% CI)	
Lower back	97	4.1	49	4.1	48	4.1	0.0 (-1.6 ; 1.7)	1.03 (0.68 ; 1.54)
Buttock	77	3.2	39	3.3	38	3.2	0.1 (-1.5 ; 1.6)	1.04 (0.66 ; 1.63)
Hip	115	4.8	57	4.8	58	4.9	-0.1 (-1.9 ; 1.8)	0.99 (0.68 ; 1.44)
Groin	65	2.7	35	2.9	30	2.5	0.4 (-1.0 ; 1.8)	1.18 (0.72 ; 1.94)
Ventral thigh	44	1.9	18	1.5	26	2.2	-0.7 (-1.9 ; 0.5)	0.69 (0.38 ; 1.27)
Dorsal thigh	83	3.5	33	2.8	50	4.2	-1.4 (-3.0 ; 0.1)	0.65 (0.42 ; 1.02)
Knee	277	11.6	129	10.8	148	12.5	-1.7 (-4.3 ; 1.0)	0.86 (0.67 ; 1.11)
Shin	75	3.2	36	3.0	39	3.3	-0.3 (-1.8 ; 1.2)	0.93 (0.58 ; 1.47)
Calf	156	6.6	82	6.9	74	6.3	0.6 (-1.5 ; 2.7)	1.13 (0.81 ; 1.56)
Achilles tendon	100	4.2	52	4.3	48	4.1	0.2 (-1.4 ; 2.0)	1.10 (0.73 ; 1.64)
Ankle	114	4.8	62	5.2	52	4.4	0.8 (-1.0 ; 2.6)	1.22 (0.83 ; 1.78)
Foot	140	5.9	71	5.9	69	5.8	0.1 (-1.9 ; 2.1)	1.04 (0.74 ; 1.47)
Toe	26	1.1	12	1.0	14	1.2	-0.2 (-1.2 ; 0.7)	0.86 (0.40 ; 1.87)

[a] Control group is reference
