# Supplementary material for: Online multifactorial prevention programme has no effect on the number of running-related injuries: a randomised controlled trial
Source: Br J Sports Med. 2019 Apr 6;53(23):1479–85. doi: 10.1136/bjsports-2018-099744 (PMC6900232; doi:10.1136/bjsports-2018-099744)
Supplement: Supplementary data [file bjsports-2018-099744supp002.pdf]

Lower leg injuries in runners who indicated that used the biomechanics section (N=273) compared to the control group (N=1182)

Intervention group		Control group		Difference	OR (95% CI) [a]	Risk ratio (95% CI)
N	%	N	%	% (95% CI)		
77	28.2	178	15.1	13.2 (7.7 ; 19.1)	1.74 (1.28 ; 2.37)	1.87 (1.48 ; 2.36)

[a] Control group is reference
